# Supplementary material for: Error-related signaling in nucleus accumbens D2 receptor-expressing neurons guides inhibition-based choice behavior in mice
Source: Nat Commun. 2023 Apr 21;14:2284. doi: 10.1038/s41467-023-38025-3 (PMC10121661; doi:10.1038/s41467-023-38025-3)
Supplement: Supplementary file 1 — Supplementary Information [file 41467_2023_38025_MOESM1_ESM.pdf]

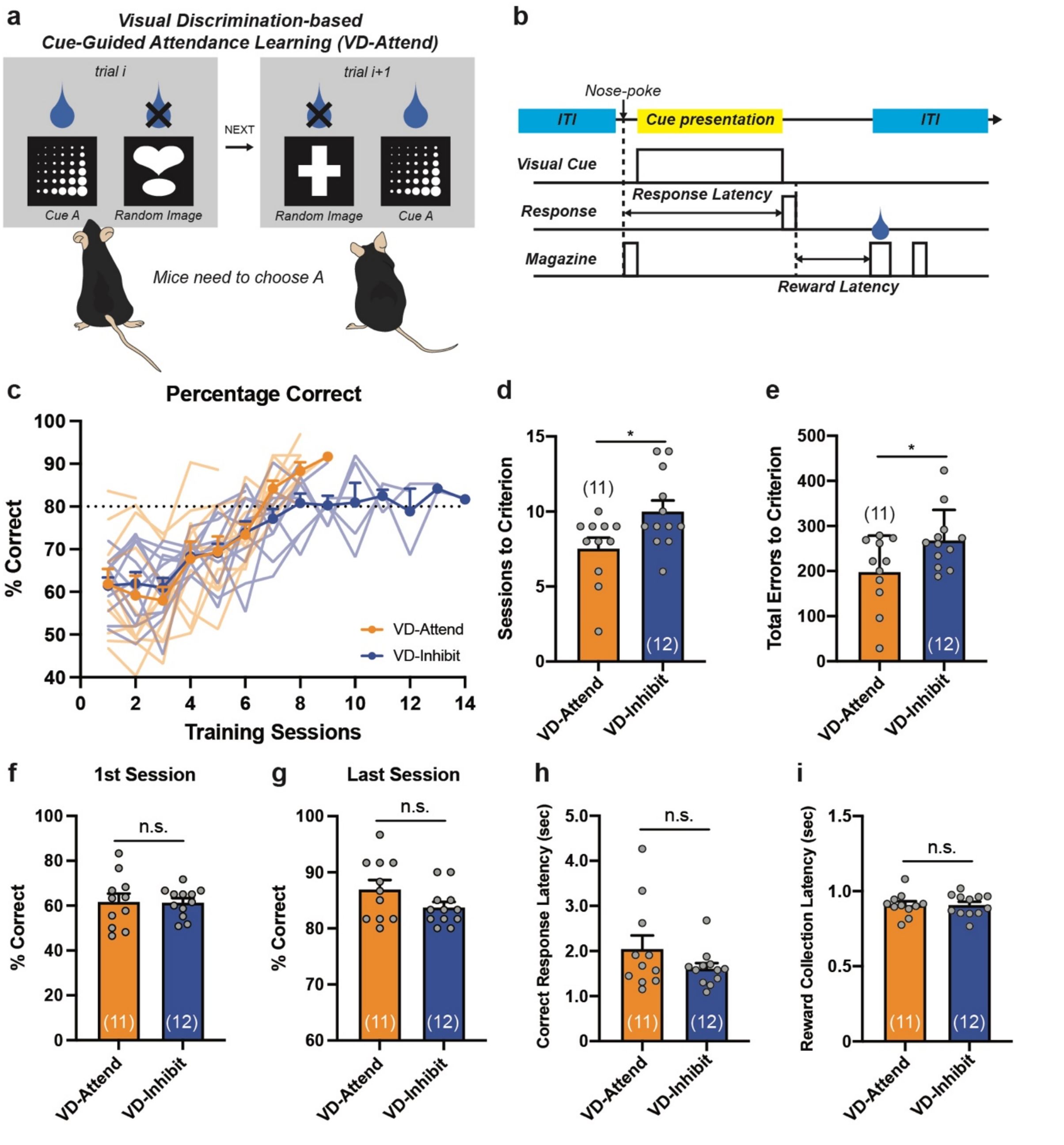

Supplementary Fig. S1. Experimental Design and Behavioral Performances of Control Task

(a) Experimental design. (b) Timeline of the task events and the definition of the behavioral parameters.
(c) The percentage of correct responses in each session (n = 11 mice in VD-Attend; n = 12 mice in VD-Inhibit). (d) The number of sessions taken to reach the criterion of the VD-Inhibit was higher than that of the VD-Attend task (Two-sided Mann Whitney test, *p = 0.0428). (e) Total errors taken to reach the criterion of the VD-Inhibit was higher than that of the VD-Attend task (Two-sided unpaired t-test, $t_{21} = 2.288$, *p = 0.0326). (f and g) The behavioral performances were similar between the VD-Attend and VD-Inhibit task in the first session (f, Two-sided unpaired t-test, $t_{21} = 0.7053$, p = 0.4884) and the last session (g, Two-sided unpaired t-test, $t_{21} = 1.613$, p = 0.1217). (h and i) Correct latencies were similar between the VD-Attend and VD-Inhibit tasks in the last session (h, Two-sided Mann Whitney test, p = 0.3793). Reward latencies were similar between the VD-Attend and VD-Inhibit tasks in the last session (i, Two-sided unpaired t-test, $t_{21} = 0.007948$, p = 0.9937). Data are presented as mean ± SEM. The numbers of mice are shown in parentheses.

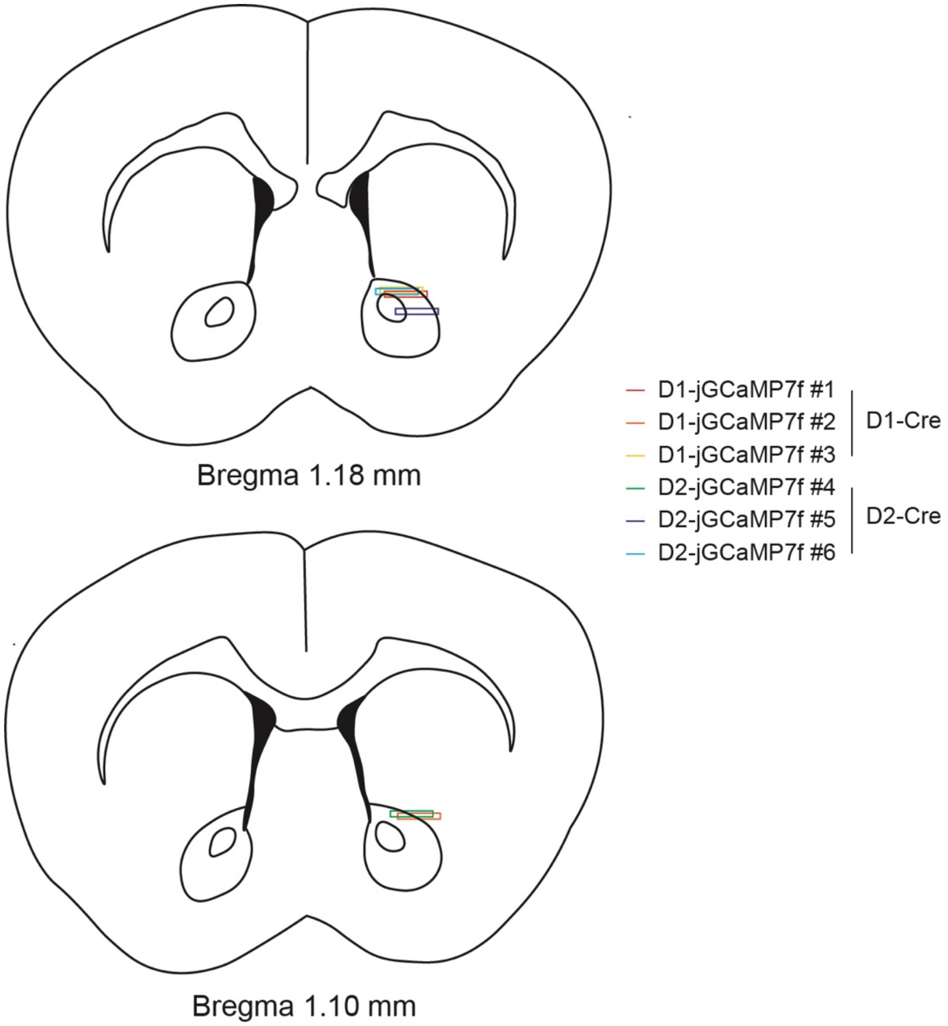

Supplementary Fig. S2. GRIN Lens Placements
Histology of GRIN lens placements.

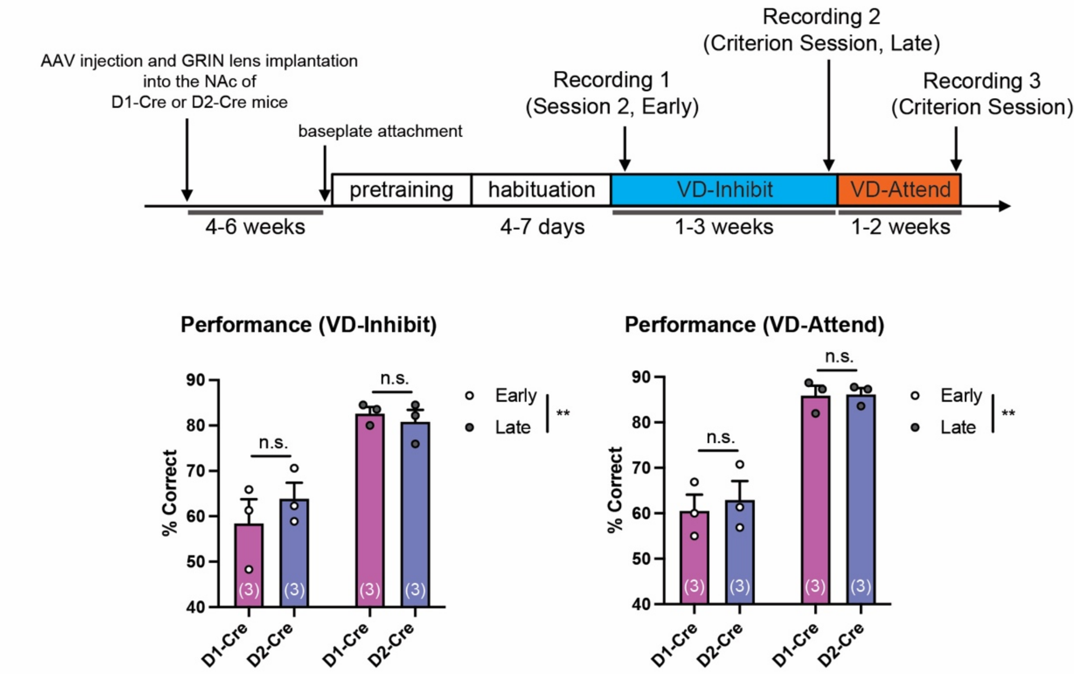

Supplementary Fig. S3. Experimental Timeline of Calcium Imaging

Experimental timeline (Top) and the behavioral performance of early and late D1-Cre and D2-Cre mice (Bottom). Behavioral performances of the VD-Inhibit task improved through learning (Two-way RM-ANOVA with Sidak correction, Learning effects, $F_{1,4} = 22.45$, $^{**}p = 0.0090$; Early, $p = 0.5112$; Late, $p = 0.9233$).

Behavioral performances of the VD-Attend task improved through learning (Two-way RM-ANOVA with Sidak correction, Learning effects, $F_{1,4} = 42.25$, $^{**}p = 0.0029$; Early, $p = 0.8264$; Late, $p = 0.9980$). Data are presented as mean ± SEM. The numbers of mice are shown in parentheses.

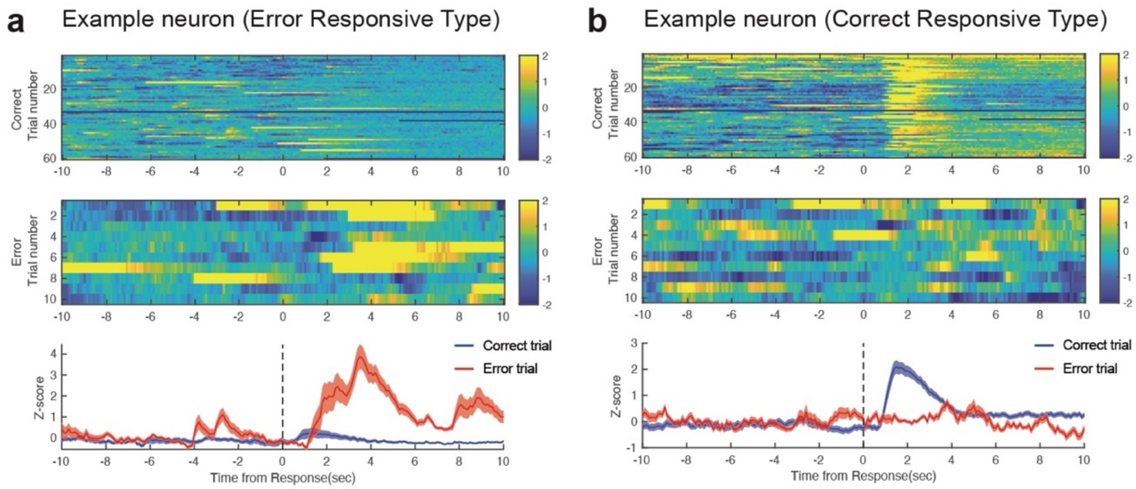

Supplementary Fig. S4. Representative Neuronal activities of an Identified Neuron

Averaged traces of two example neurons showing activity changes during (a) error or (b) correct trials. Trial-by-trial (Top) and averaged (Bottom) responses of the neurons are shown in correct and error trials. Data are presented as mean ± SEM.

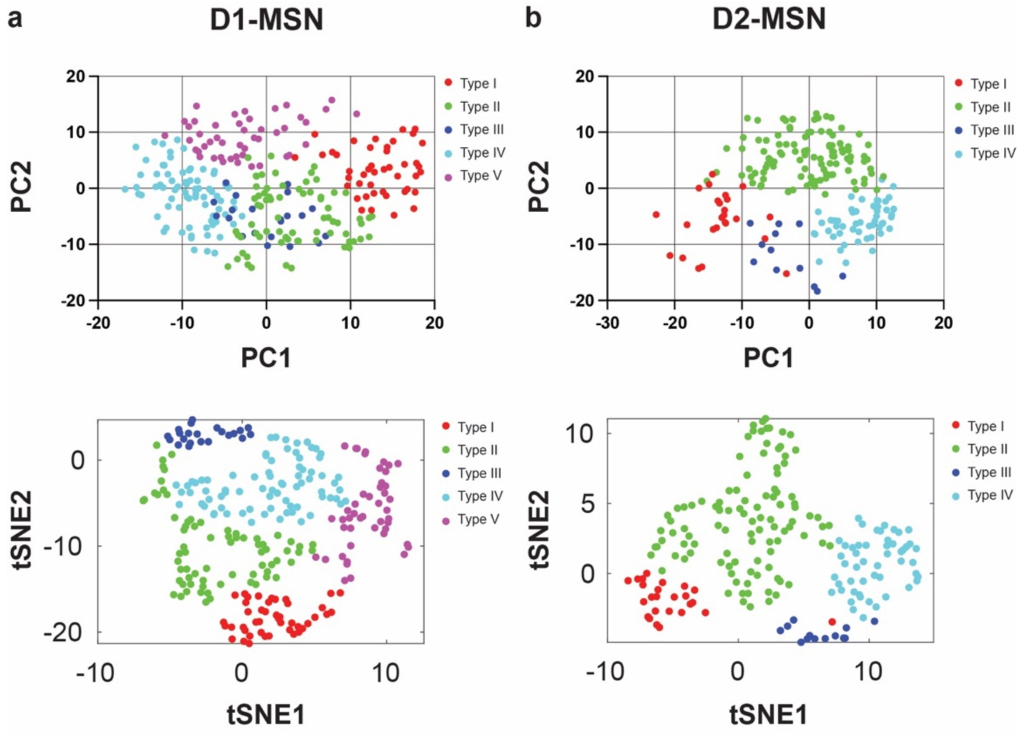

Supplementary Fig. S5. The first two principal components (PC) and t-distributed Stochastic Neighbor Embedding (tSNE) of D1- and D2-MSNs

(a) The first two PC (Top) and tSNE (Bottom) of D1-MSNs, colored by hierarchical clustering. (b) The first two PC (Top) and tSNE (Bottom) of D2-MSNs, colored by hierarchical clustering.

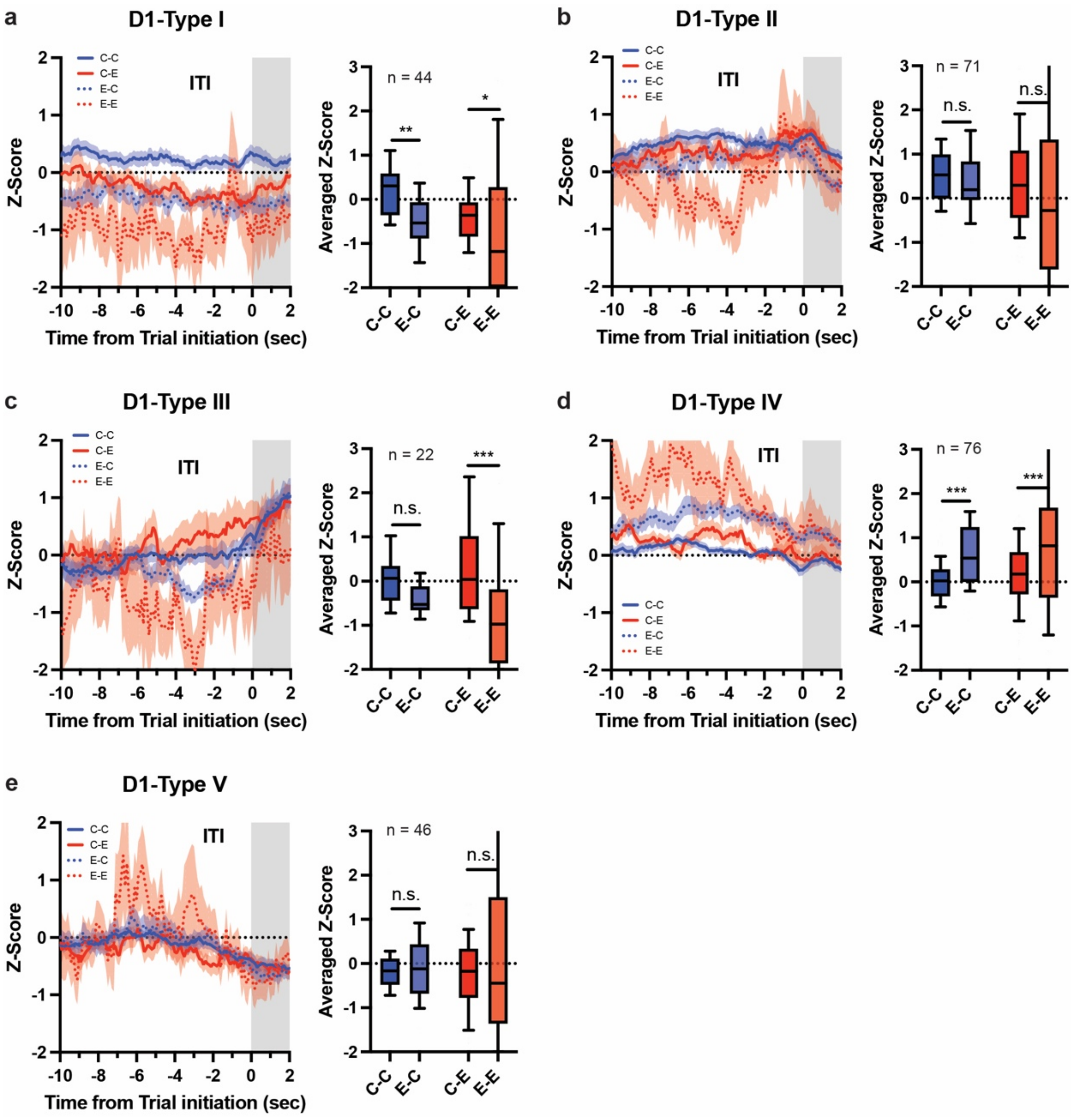

Supplementary Fig. S6. Previous Trial History Effect on the Neural Activities of D1-MSNs

(a-e) Averaged traces of D1-MSN cell types in correct trials after correct responses (solid blue line) and after error responses (dotted blue line) and in error trials after correct responses (solid red line) and after error responses (dotted red line) (Left). Averaged Z-score during ITI (-5-0 sec from trial onset) (Right, Two-way RM ANOVA with Sidak correction) for Type I (a, C-C vs E-C, **p = 0.0044; C-E vs E-E, *p = 0.0376, n = 44 cells), Type II (b, C-C vs E-C, p = 0.7401; C-E vs E-E, p = 0.1384, n = 71 cells), Type III (c, C-C vs E-C, p = 0.2627; C-E vs E-E, ***p = 0.0003, n = 22 cells), Type IV (d, C-C vs E-C, ***p = 0.0005; C-E vs E-E, ***p = 0.0010, n = 76 cells), and Type V (e, C-C vs E-C, p = 0.9649; C-E vs E-E, p = 0.3415, n = 46 cells). Data are presented as mean ± SEM. In the box plots, the center line denotes the median, the box boundaries mark the interquartile range and the whiskers extend to the 10th to 90th percentiles.

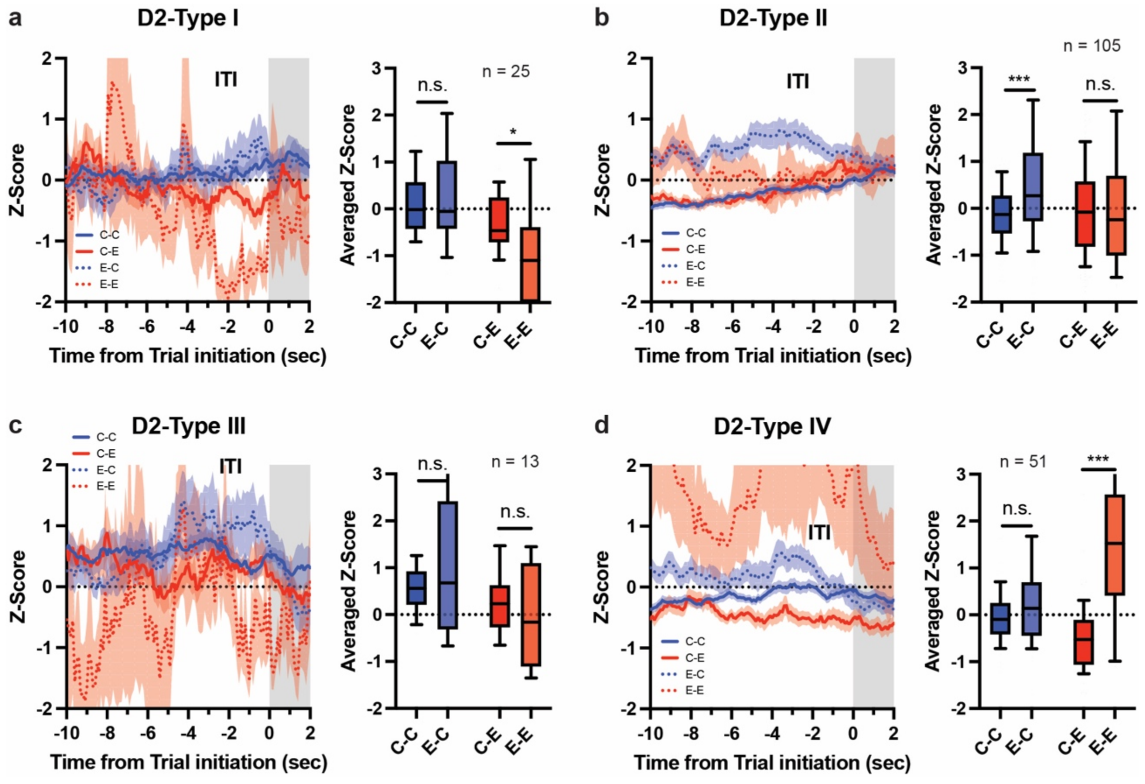

Supplementary Fig. S7. Previous Trial History Effect on the Neural Activities of D2-MSNs

(a-d) Averaged traces of D2-MSN cell types in correct trials after correct responses (solid blue line) and after error responses (dotted blue line) and in error trials after correct responses (solid red line) and after error responses (dotted red line) (Left). Averaged Z-score during ITI (-5-0 sec from trial onset) (Right, Two-way RM ANOVA with Sidak correction) for Type I (a, C-C vs E-C, $p = 0.8078$; C-E vs E-E, *$p = 0.0487$, $n = 25$ cells), Type II (b, C-C vs E-C, ***$p < 0.0001$; C-E vs E-E, $p = 0.9458$, $n = 105$ cells), Type III (c, C-C vs E-C, $p = 0.3668$; C-E vs E-E, $p = 0.9545$, $n = 13$ cells), and Type IV (d, C-C vs E-C, $p = 0.2985$; C-E vs E-E, ***$p < 0.0001$, $n = 51$ cells). Data are presented as mean ± SEM. In the box plots, the center line denotes the median, the box boundaries mark the interquartile range and the whiskers extend to the 10th to 90th percentiles.

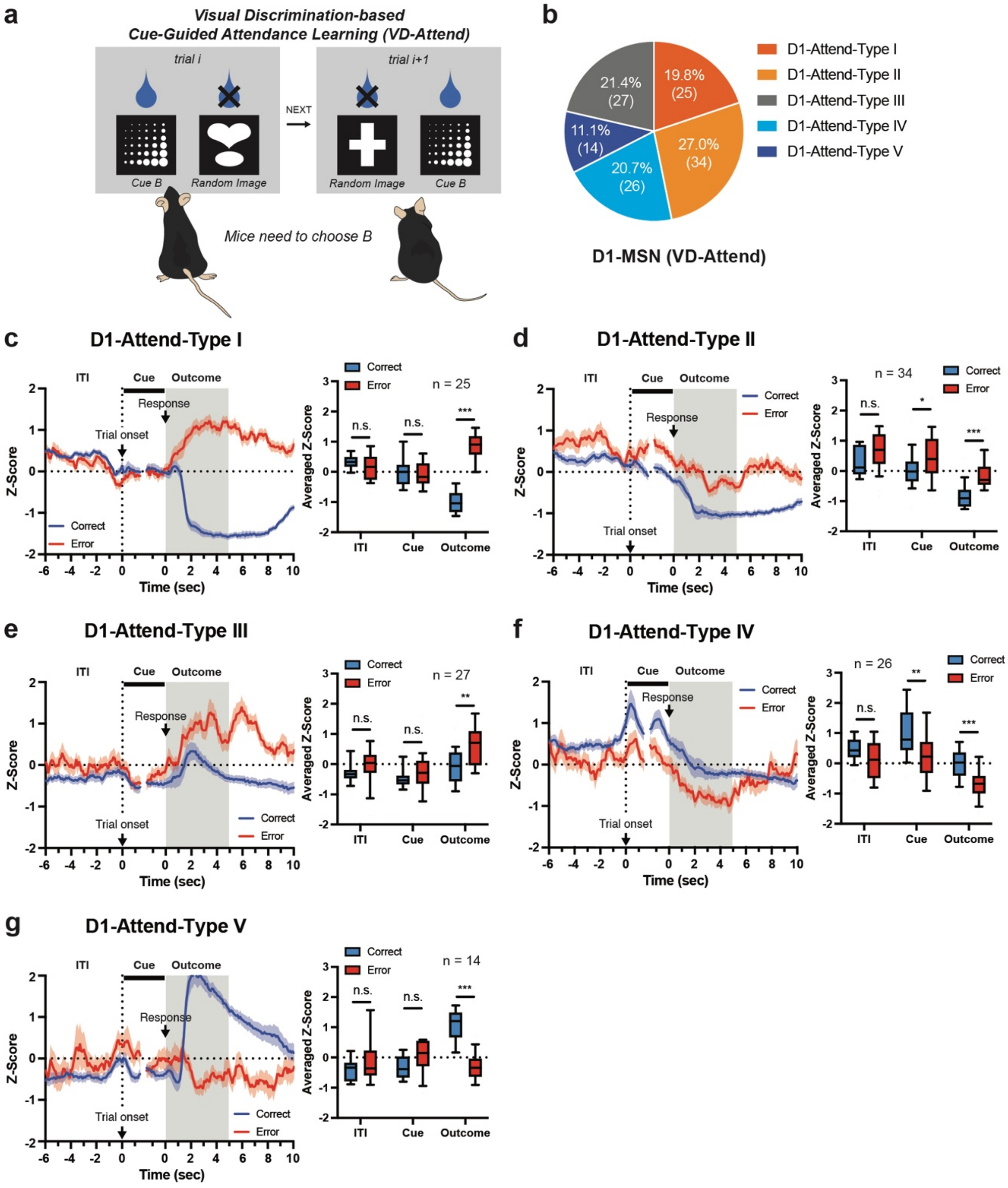

Supplementary Fig. S8. Neural Activity of D1-MSN in Mice Performing the VD-Attend Task

(a) Experimental design. (b) Proportion of each type of cells in D1-MSN in mice performing the VD-Attend task. (c-g) Averaged traces of D1-MSN cell types in correct and error trials (Left) and averaged Z-score during ITI (-5-0 sec from trial onset), Cue (0-1.5 sec from trial onset and -1.5-0 sec from a response), and Outcome (0-5 sec from response) period (Right, Two-way RM ANOVA with Sidak correction) for Type I (c, ITI, $p = 0.1997$; Cue, $p = 0.4712$; Outcome, ***$p < 0.0001$, n = 25 cells), Type II (d, ITI, $p = 0.0503$; Cue, *$p = 0.0176$; Outcome, ***$p < 0.0001$, n = 34 cells), Type III (e, ITI, $p = 0.2185$; Cue, $p = 0.5759$; Outcome, **$p = 0.0029$, n = 27 cells), Type IV (f, ITI, $p = 0.0881$; Cue, **$p = 0.0032$; Outcome, ***$p = 0.0007$, n = 26 cells), and Type V (g, ITI, $p = 0.4940$; Cue, $p = 0.1908$; Outcome, ***$p < 0.0001$, n = 14 cells). Data are presented as mean ± SEM. In the box plots, the center line denotes the median, the box boundaries mark the interquartile range and the whiskers extend to the 10th to 90th percentiles.

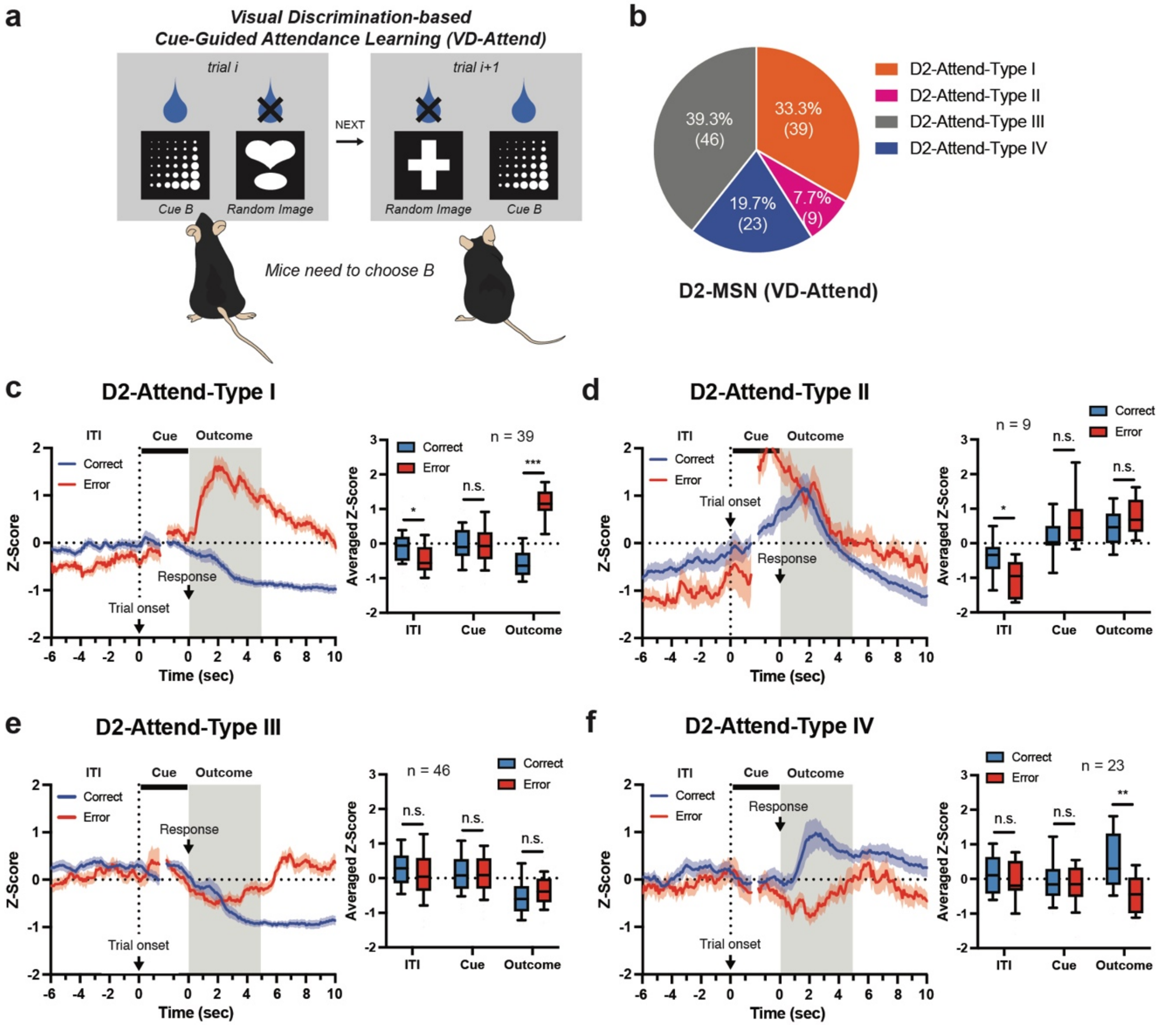

Supplementary Fig. S9. Neural Activity of D2-MSN in Mice Performing the VD-Attend Task

(a) Experimental design.
(b) Proportion of each type of cells in D2-MSN in mice performing the VD-Attend task.
(c-f) Averaged traces of D2-MSN cell types in correct and error trials (Left) and averaged Z-score during ITI (-5-0 sec from trial onset), Cue (0-1.5 sec from trial onset and -1.5-0 sec from a response), and Outcome (0-5 sec from response) period (Right, Two-way RM ANOVA with Sidak correction) for Type I (c, ITI, *p = 0.0271; Cue, p = 0.9992; Outcome, ***p < 0.0001, n = 39 cells), Type II (d, ITI, *p = 0.0350; Cue, p = 0.1207; Outcome, p = 0.3324, n = 9 cells), Type III (e, ITI, p = 0.4915; Cue, p = 0.9887; Outcome, p = 0.5217, n = 46 cells), and Type IV (f, ITI, p = 0.4210; Cue, p = 0.8437; Outcome, **p = 0.0021, n = 23 cells). Data are presented as mean ± SEM. In the box plots, the center line denotes the median, the box boundaries mark the interquartile range and the whiskers extend to the 10th to 90th percentiles.

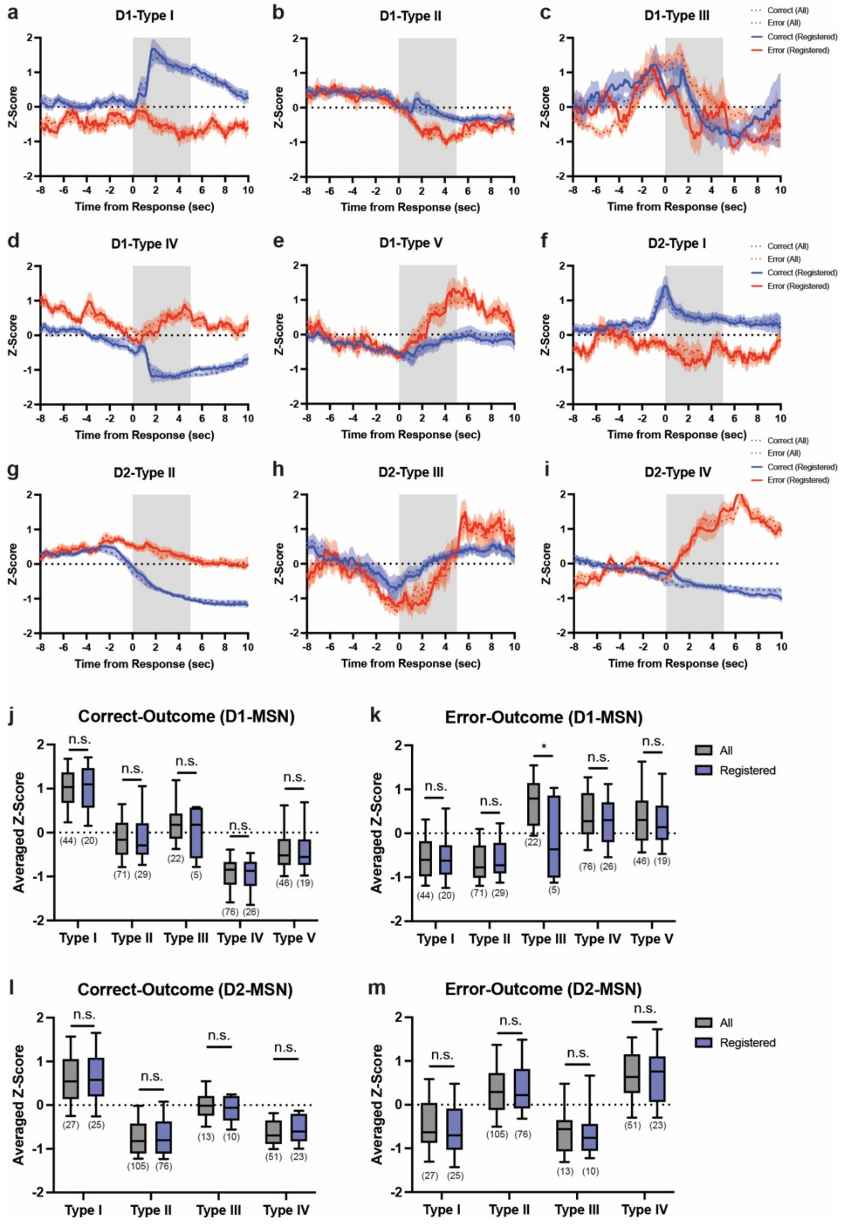

Supplementary Fig. S10. The Neural Activity of the Registered cells Recapitulates the Neural Activity of the Original Cluster.

(a-i) Comparison of population averaged traces of original (dotted line) and cell-registered clusters (solid line). (j) Comparison of average neural activity for each cluster of D1-MSNs on correct trials (Two-way RM ANOVA with Sidak correction, Type I, $p > 0.9999$, $n = 44$ cells in All and $n = 20$ cells in Registered; Type II, $p = 0.9976$, $n = 71$ cells in All and $n = 29$ cells in Registered; Type III, $p = 0.9297$, $n = 22$ cells in All and $n = 5$ cells in Registered; Type IV, $p = 0.9987$, $n = 76$ cells in All and $n = 26$ cells in Registered; Type V, $p = 0.9999$, $n = 46$ cells in All and $n = 19$ cells in Registered). (k) Comparison of average neural activity for each cluster of D1-MSNs on error trials (Two-way RM ANOVA with Sidak correction, Type I, $p > 0.9999$; Type II, $p = 0.9558$; Type III, $^*p = 0.0195$; Type IV, $p = 0.9603$; Type V, $p = 0.9871$). (l) Comparison of average neural activity for each cluster of D2-MSNs on correct trials (Two-way RM ANOVA with Sidak correction, Type I, $p = 0.9881$, $n = 27$ cells in All and $n = 25$ cells in Registered; Type II, $p = 0.9836$, $n = 105$ cells in All and $n = 76$ cells in Registered; Type III, $p = 0.9960$, $n = 13$ cells in All and $n = 10$ cells in Registered; Type IV, $p = 0.9406$, $n = 51$ cells in All and $n = 23$ cells in Registered). (m) Comparison of average neural activity for each cluster of D2-MSNs on error trials (Two-way RM ANOVA with Sidak correction, Type I, $p = 0.9624$; Type II, $p = 0.9668$; Type III, $p = 0.9995$; Type IV, $p > 0.9999$). Data are presented as mean ± SEM. In the box plots, the center line denotes the median, the box boundaries mark the interquartile range and the whiskers extend to the 10th to 90th percentiles. The numbers of cells are shown in parentheses.

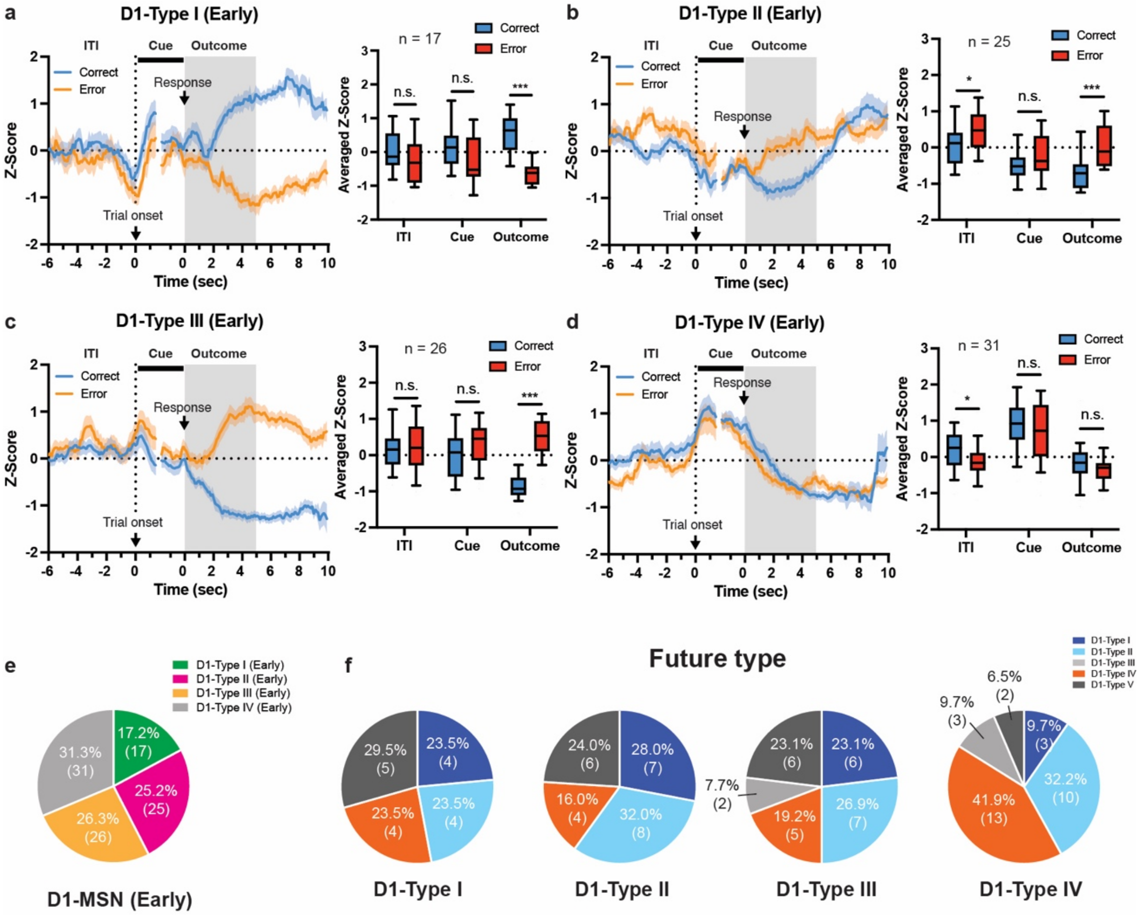

Supplementary Fig. S11. Cluster Type Transition of D1-MSNs from Early to Late

(a-d) Population averaged traces of D1-MSNs cell types in correct and error trials in the early stage of learning (Left). Averaged Z-score during the ITI (-5-0 sec from trial onset), Cue (0-1.5 sec from trial onset and -1.5-0 sec from a response), and Outcome (0-5 sec from response) period (Right; Two-way RM ANOVA with Sidak correction) for Type I (a, ITI, $p = 0.3517$; Cue, $p = 0.0533$; Outcome, ***$p < 0.0001$, n = 17 cells), Type II (b, ITI, *$p = 0.0197$; Cue, $p = 0.2492$; Outcome, ***$p < 0.0001$, n = 25 cells), Type III (c, ITI, $p = 0.8242$; Cue, $p = 0.1487$; Outcome, ***$p < 0.0001$, n = 26 cells), and Type IV (d, ITI, *$p = 0.0457$; Cue, $p = 0.4297$; Outcome, $p = 0.4582$, n = 31 cells). (e) Proportion of each type of D1-MSN cell in the early stage of learning. (f) Proportion of cluster types that each cluster will become in the future. Data are presented as mean ± SEM. In the box plots, the center line denotes the median, the box boundaries mark the interquartile range and the whiskers extend to the 10th to 90th percentiles.

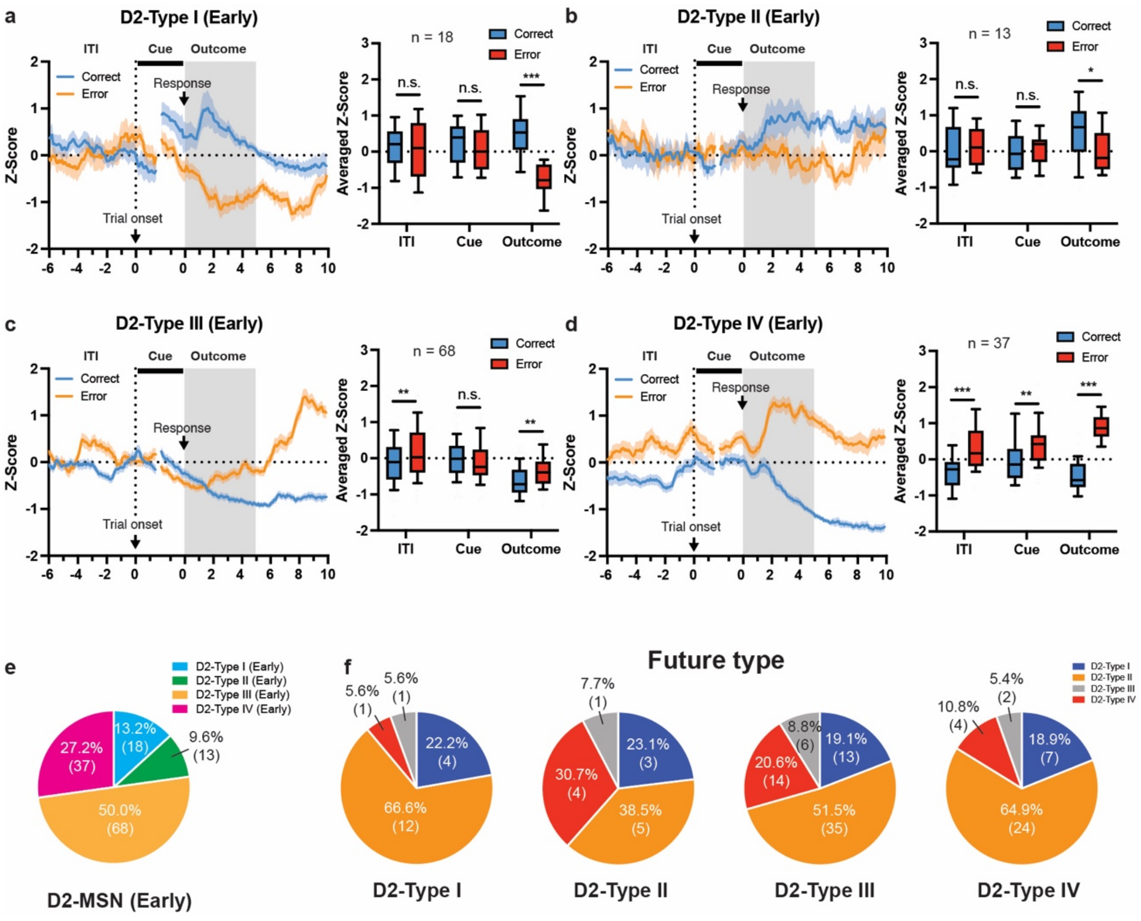

Supplementary Fig. S12. Cluster Type Transition of D2-MSNs from Early to Late

(a-d) Population averaged traces of D2-MSNs cell types in correct and error trials in the early stage of learning (Left). Averaged Z-score during the ITI (-5-0 sec from trial onset), Cue (0-1.5 sec from trial onset and -1.5-0 sec from a response), and Outcome (0-5 sec from response) period (Right; Two-way RM ANOVA with Sidak correction) for Type I (a, ITI, $p = 0.9332$; Cue, $p = 0.7445$; Outcome, ***$p < 0.0001$, n = 18 cells), Type II (b, ITI, $p = 0.9717$; Cue, $p = 0.9569$; Outcome, *$p = 0.0259$, n = 13 cells), Type III (c, ITI, **$p = 0.0054$; Cue, $p = 0.5169$; Outcome, **$p = 0.0018$, n = 68 cells), and Type IV (d, ITI, ***$p < 0.0001$; Cue, **$p = 0.0028$; Outcome, ***$p < 0.0001$, n = 37 cells). (e) Proportion of each type of D2-MSN cell in the early stage of learning. (f) Proportion of cluster types that each cluster will become in the future. Data are presented as mean ± SEM. In the box plots, the center line denotes the median, the box boundaries mark the interquartile range and the whiskers extend to the 10th to 90th percentiles.

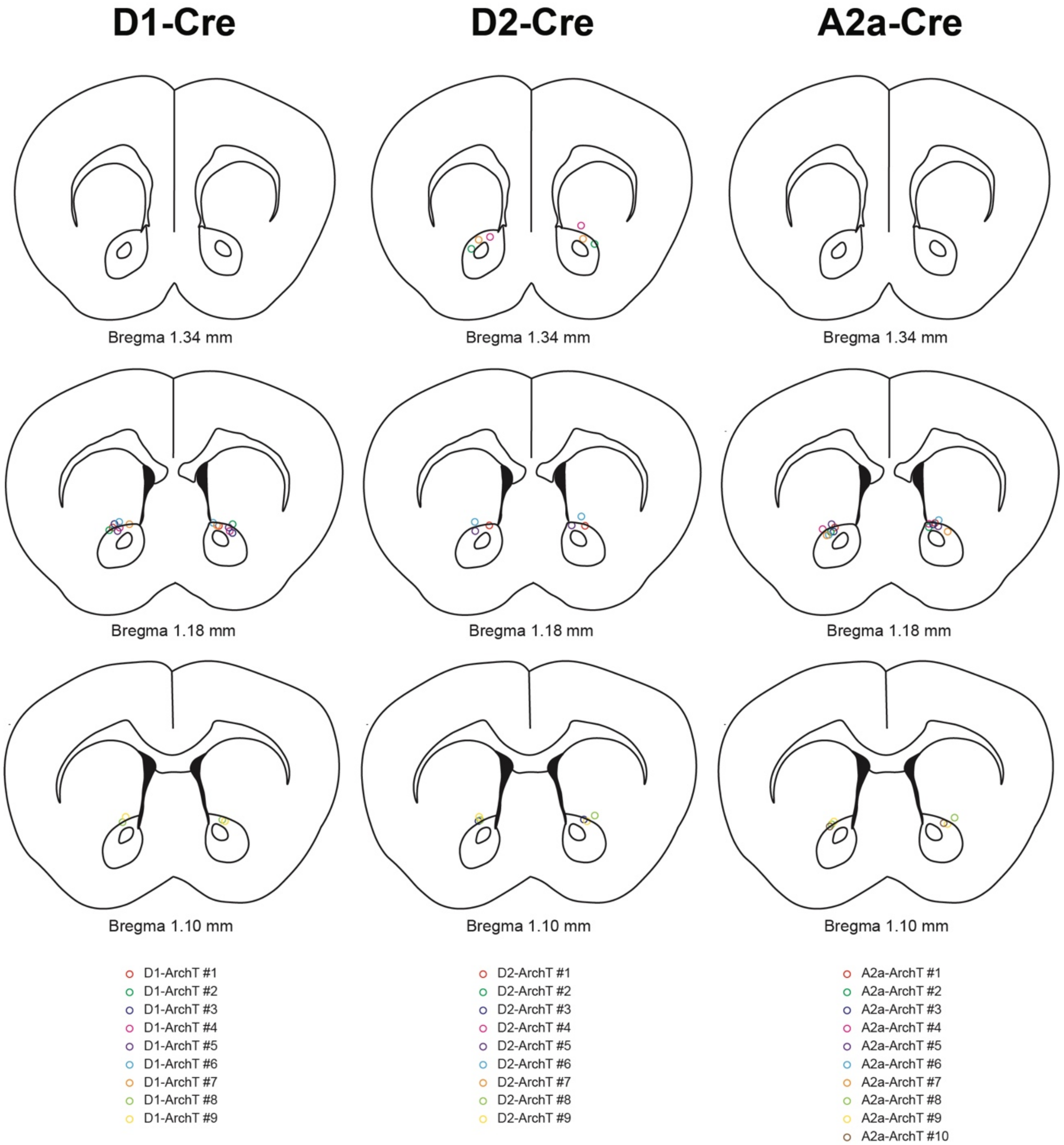

Supplementary Fig. S13. Optic Fiber Placements
Histology of optic fiber placements for D1-, D2-, and A2a-Cre mice.

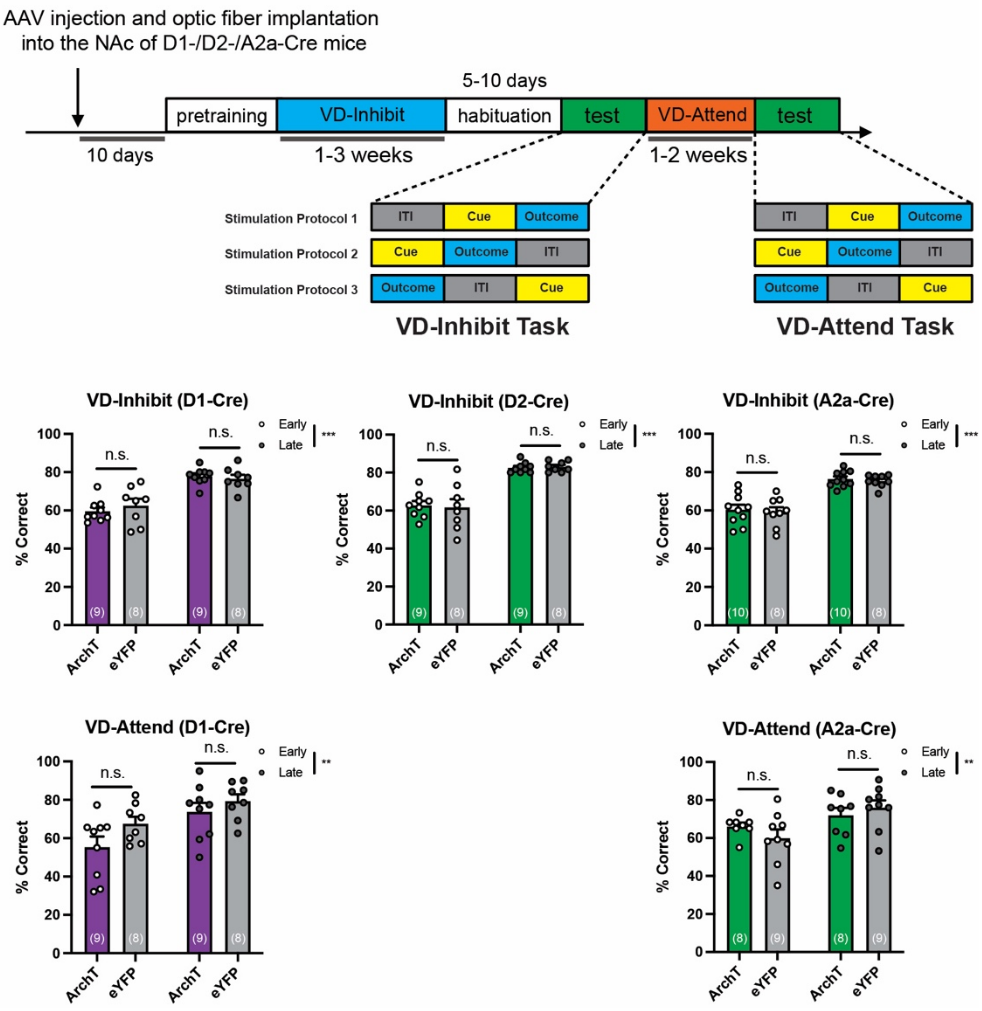

Supplementary Fig. S14. Experimental Timeline of Optogenetic Manipulation

Experimental timeline (Top) and the behavioral performance of mice with optic fiber during the training (Bottom). There was no difference in the performance of the VD-Inhibit task between the ArchT and eYFP groups. (D1-Cre, Two-way RM-ANOVA with Sidak correction, Learning effects, $F_{1,15} = 33.87$, ***$p < 0.0001$; Early, $p = 0.5951$; Late, $p = 0.9019$; D2-Cre, Two-way RM-ANOVA with Sidak correction, Learning effects, $F_{1,15} = 69.52$, ***$p < 0.0001$; Early, $p = 0.9441$; Late, $p = 0.9849$; A2a-Cre, Two-way RM-ANOVA with Sidak correction, Learning effects, $F_{1,17} = 78.63$, ***$p < 0.0001$; Early, $p = 0.8933$; Late, $p = 0.9260$). There was no difference in the performance of the VD-Attend task between the ArchT and eYFP groups. (D1-Cre, Two-way RM-ANOVA with Sidak correction, Learning effects, $F_{1,15} = 16.25$, **$p = 0.0011$; Early, $p = 0.1213$; Late, $p = 0.6137$; A2a-Cre, Two-way RM-ANOVA with Sidak correction, Learning effects, $F_{1,15} = 9.752$, **$p = 0.0070$; Early, $p = 0.4358$; Late, $p = 0.7021$). Data are presented as mean ± SEM. The numbers of mice are shown in parentheses.

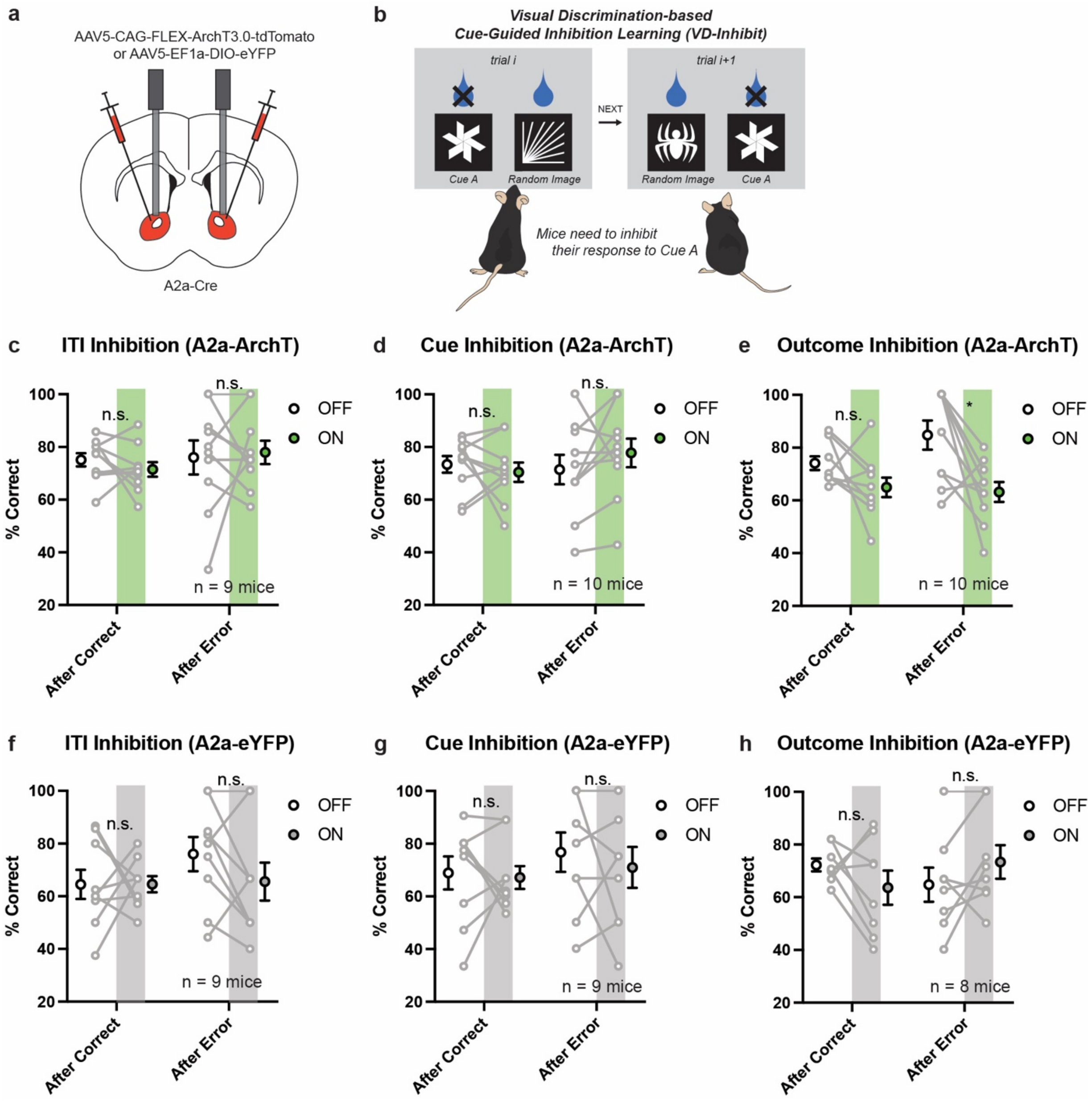

Supplementary Fig. S15. Post-Error Activation of A2A Neurons is Necessary for Inhibition-Based Choice Behavior

(a) Schematic of viral injection and optic fiber implantation. (b) Experimental design. (c and d) Optogenetic suppression of A2a-expressing neurons (D2-MSNs) in the NAc during the ITI (c) or Cue period (d) did not affect the behavioral performance of ArchT mice in the next trial (c, Two-way RM-ANOVA with Sidak correction; After Correct, $p = 0.6491$; After Error, $p = 0.7056$; d, Two-way RM-ANOVA with Sidak correction; After Correct, $p = 0.7053$; After Error, $p = 0.5795$). (e) Optogenetic suppression of A2a-expressing neurons in the NAc during the Outcome period of error trials impaired the behavioral performance of ArchT mice in the next trial (Two-way RM-ANOVA with Sidak correction; After Correct, $p = 0.2765$; After Error, $^{*}p = 0.0257$). (f-h) Optical stimulation in the NAc during the ITI (f), Cue (g), or Outcome periods (h) did not affect the behavioral performance of ArchT mice in the next trial (f, Two-way RM-ANOVA with Sidak correction; After Correct, $p > 0.9999$; After Error, $p = 0.3147$; g, Two-way RM-ANOVA with Sidak correction; After Correct, $p = 0.9304$; After Error, $p = 0.4832$; h, Two-way RM-ANOVA with Sidak correction; After Correct, $p > 0.9999$; After Error, $p > 0.9999$). Data are presented as mean ± SEM.

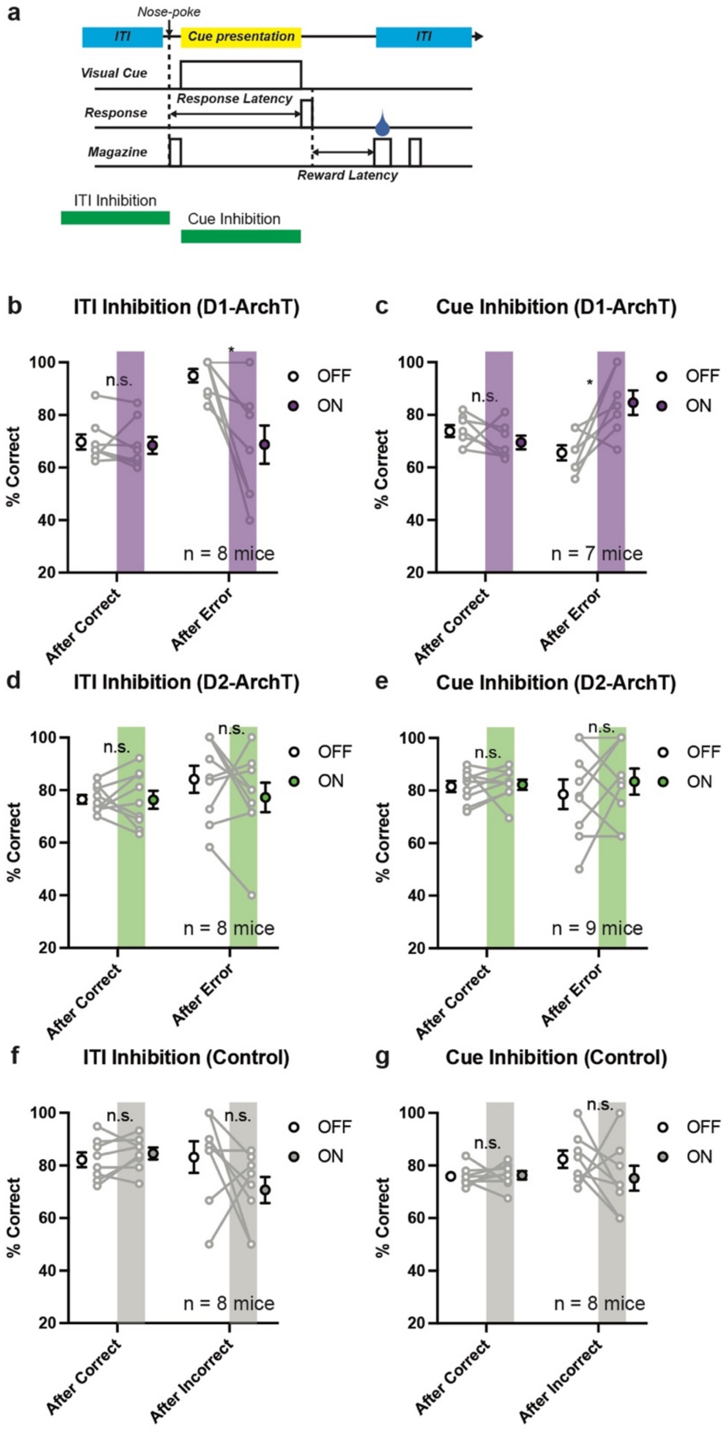

Supplementary Fig. S16. Optogenetic Suppression during the ITI and Cue Periods

(a) Schematic of optical stimulation protocol. (b) Optogenetic suppression of D1-MSNs in the NAc during the ITI period after an error response decreased the performance of ArchT mice (Two-way RM-ANOVA with Sidak correction; After Correct, $p = 0.8655$; After Error, *$p = 0.0195$). (c) Optogenetic suppression of D1-MSNs in the NAc during the Cue period after an error response improved the performance of ArchT mice (Two-way RM-ANOVA with Sidak correction; After Correct, $p = 0.4624$; After Error, *$p = 0.0487$). (d and e) Optogenetic suppression of D2-MSNs in the NAc during the ITI (d) or Cue period (e) did not affect the performance of ArchT mice in the next trial (d, Two-way RM-ANOVA with Sidak correction; After Correct, $p = 0.9945$; After Error, $p = 0.5433$; e, Two-way RM-ANOVA with Sidak correction; After Correct, $p = 0.9760$; After Error, $p = 0.3471$). (f and g) LED delivery to the NAc during the ITI (f) and Cue period (g) did not affect the performance of control (eYFP) mice (f, Two-way RM-ANOVA with Sidak correction; After Correct, $p = 0.9265$; After Error, $p = 0.1910$; g, Two-way RM-ANOVA with Sidak correction; After Correct, $p = 0.9961$; After Error, $p = 0.3302$). Data are presented as mean ± SEM.

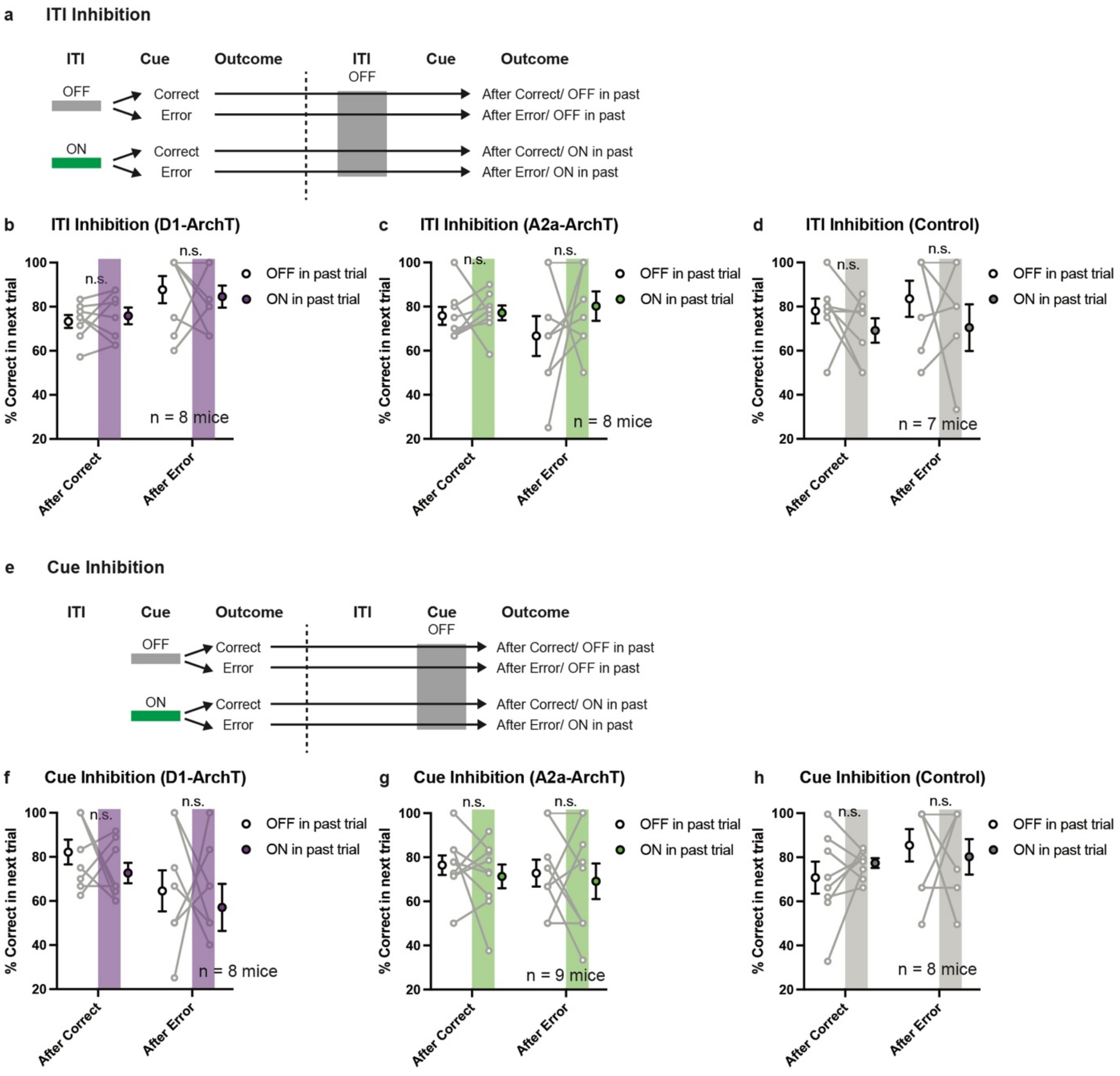

Supplementary Fig. S17. Optogenetic Suppression of D1- and D2-MSNs during the ITI or Cue Period Did Not Affect the Performance on Subsequent Trials

(a) Schematic of optical stimulation protocol for ITI inhibition. (b) Optogenetic suppression of D1-MSNs in the NAc during the ITI period did not affect the performance of ArchT mice in the next trial (Two-way RM-ANOVA with Sidak correction; After Correct, $p = 0.8720$; After Error, $p = 0.8168$). (c) Optogenetic suppression of D2-MSNs in the NAc during the ITI period did not affect the performance of ArchT mice in the next trial (Two-way RM-ANOVA with Sidak correction; After Correct, $p = 0.9843$; After Error, $p = 0.2493$). (d) LED delivery to the NAc during the ITI period did not affect the behavioral performance of control (eYFP) mice in the next trial (Two-way RM-ANOVA with Sidak correction; After Correct, $p = 0.6186$; After Error, $p = 0.3812$). (e) Schematic of optical stimulation protocol for Cue inhibition. (f) Optogenetic suppression of D1-MSNs in the NAc during the Cue period did not affect the performance of ArchT mice in the next trial (Two-way RM-ANOVA with Sidak correction; After Correct, $p = 0.5827$; After Error, $p = 0.7072$). (g) Optogenetic suppression of D2-MSNs in the NAc during the Cue period did not affect the performance of ArchT mice in the next trial (Two-way RM-ANOVA with Sidak correction; After Correct, $p = 0.8013$; After Error, $p = 0.8890$). (h) LED delivery to the NAc during the Cue period did not affect the performance of control (eYFP) mice in the next trial (Two-way RM-ANOVA with Sidak correction; After Correct, $p = 0.8157$; After Error, $p = 0.8789$). Data are presented as mean ± SEM.

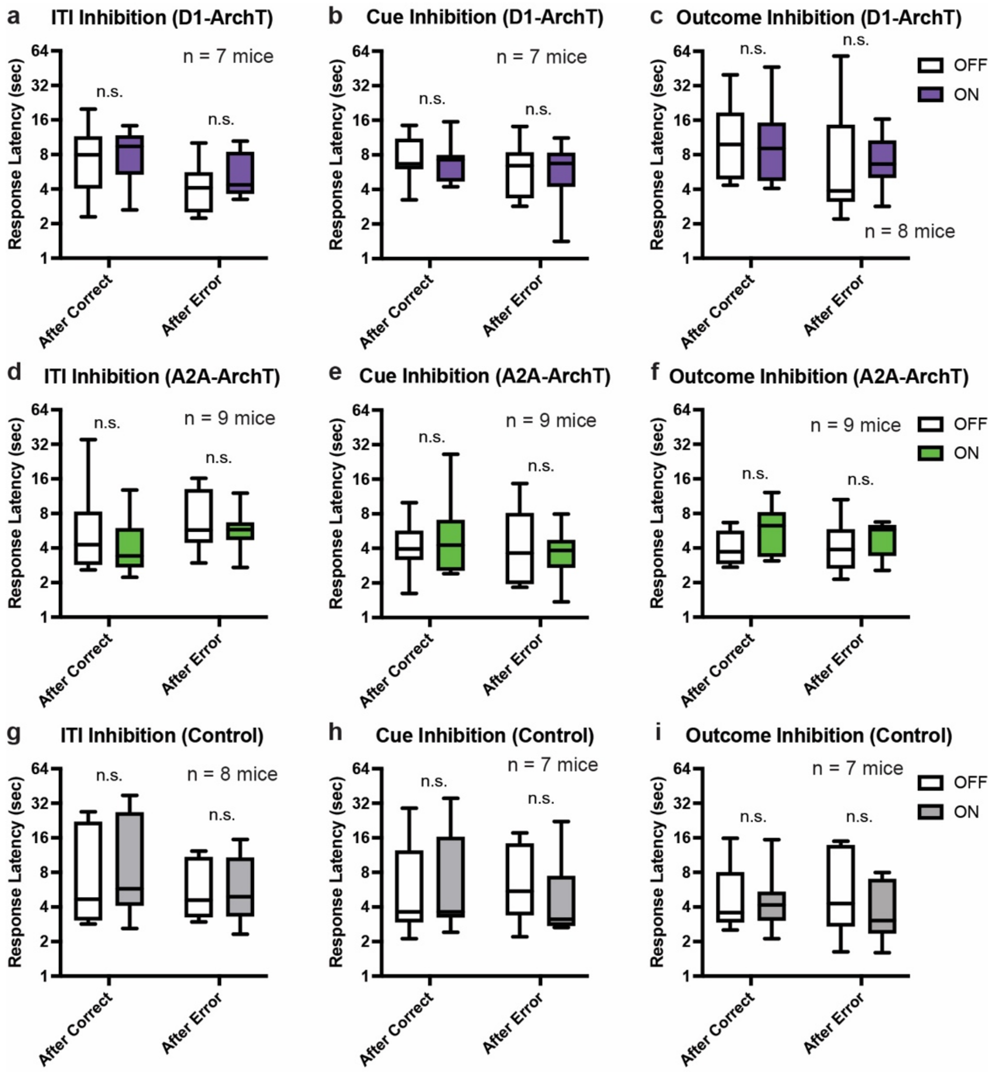

Supplementary Fig. S18. Optogenetic Suppression of D1- and D2-MSNs Did Not Affect Response Latencies

(a-c) Optogenetic suppression of D1-MSNs in the NAc during the ITI (a), Cue (b) or Outcome period (c) did not affect the response latencies of ArchT mice in the next trial (a, Two-way RM-ANOVA with Sidak correction; After Correct, $p = 0.9993$; After Error, $p = 0.9028$, $n = 7$ mice; b, Two-way RM-ANOVA with Sidak correction; After Correct, $p = 0.8118$; After Error, $p = 0.9024$, $n = 7$ mice; c, Two-way RM-ANOVA with Sidak correction; After Correct, $p = 0.9984$; After Error, $p = 0.6137$, $n = 8$ mice). (d-f) Optogenetic suppression of D2-MSNs in the NAc during the ITI (d), Cue (e) or Outcome period (f) did not affect the response latencies of ArchT mice in the next trial (d, Two-way RM-ANOVA with Sidak correction; After Correct, $p = 0.1372$; After Error, $p = 0.4538$, $n = 9$ mice; e, Two-way RM-ANOVA with Sidak correction; After Correct, $p = 0.4277$; After Error, $p = 0.6773$, $n = 9$ mice; f, Two-way RM-ANOVA with Sidak correction; After Correct, $p = 0.0570$; After Error, $p = 0.7493$, $n = 9$ mice). (g-i) LED delivery to the NAc during the ITI (g), Cue (h) or Outcome period (i) did not affect the response latencies of control (eYFP) mice in the next trial (g, Two-way RM-ANOVA with Sidak correction; After Correct, $p = 0.1757$; After Error, $p = 0.8578$, $n = 8$ mice; h, Two-way RM-ANOVA with Sidak correction; After Correct, $p = 0.4365$; After Error, $p = 0.3032$, $n = 7$ mice; i, Two-way RM-ANOVA with Sidak correction; After Correct, $p = 0.9676$; After Error, $p = 0.1701$, $n = 7$ mice). In the box plots, the center line denotes the median, the box boundaries mark the interquartile range and the whiskers extend to the 10th to 90th percentiles.

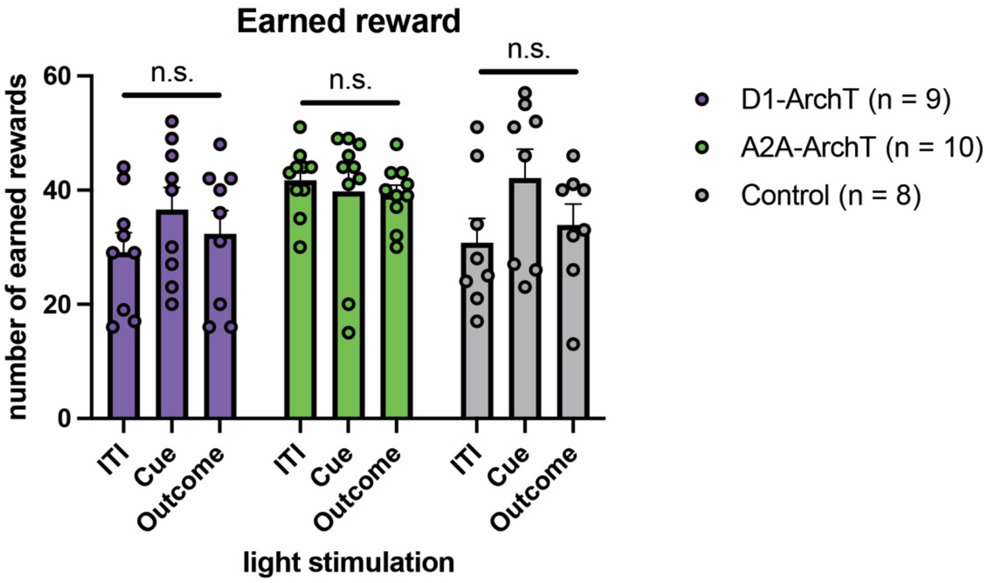

Supplementary Fig. S19. Optogenetic Suppression of D1- and D2-MSNs Did Not Affect the Number of Earned Rewards

Optogenetic suppression of D1- or D2-MSNs in the NAc during the ITI, Cue or Outcome period did not affect the number of earned rewards in ArchT mice (Two-way RM-ANOVA with Sidak correction; D1-Cre, Cue vs ITI, $p = 0.3094$; Outcome vs ITI, $p = 0.7997$; Outcome vs Cue, $p = 0.6821$; D2-Cre, Cue vs ITI, $p = 0.9171$; Outcome vs ITI, $p = 0.8610$; Outcome vs Cue, $p = 0.9914$; Control, Cue vs ITI, $p = 0.0922$; Outcome vs ITI, $p = 0.8295$; Outcome vs Cue, $p = 0.2785$). Data are presented as mean ± SEM.

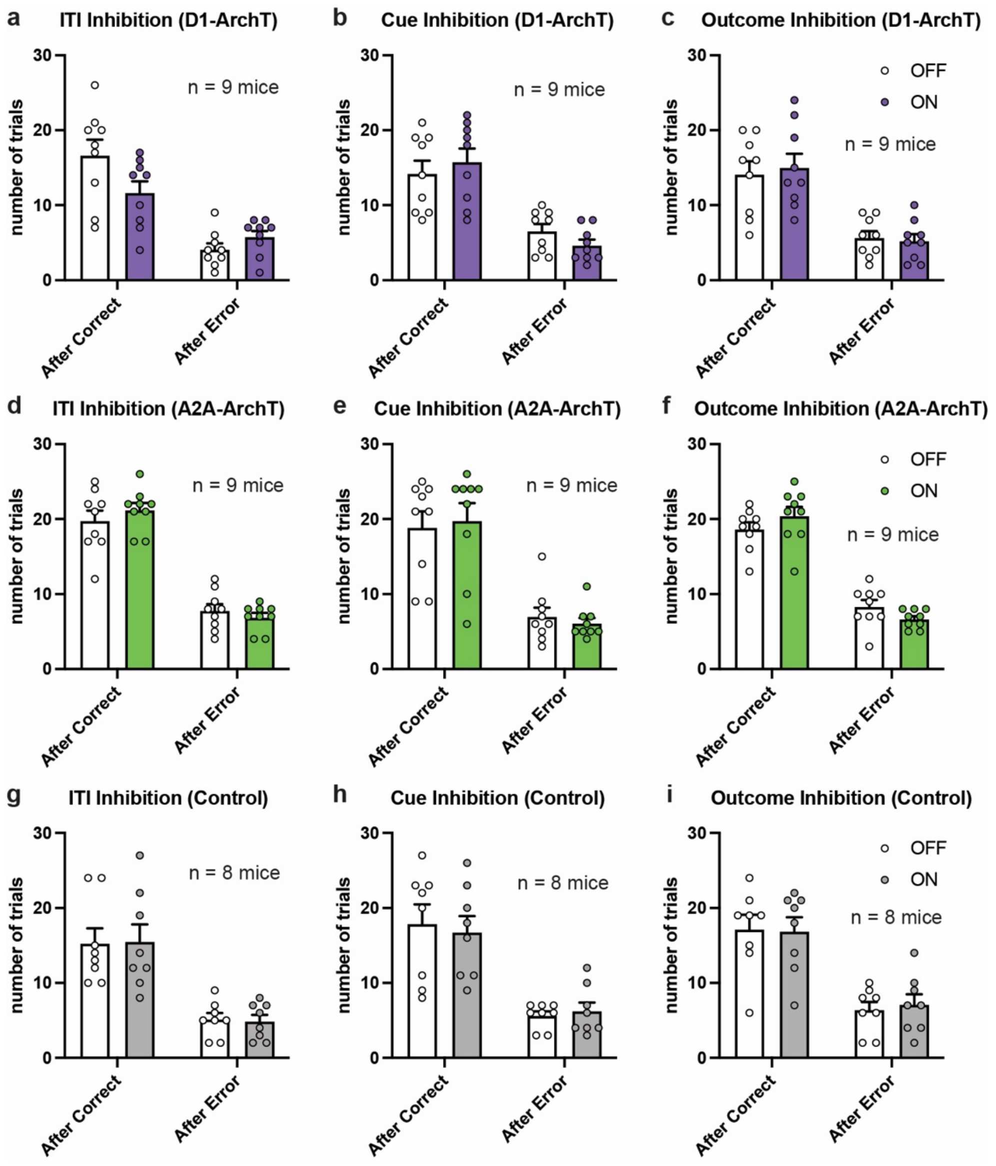

Supplementary Fig. S20. The Number of Trials for Each Group

(a-c) The number of trials for D1-ArchT. (d-f) The number of trials for D2-ArchT. (g-i) The number of trials for Control. Data are presented as mean ± SEM. The numbers of mice are shown in parentheses.

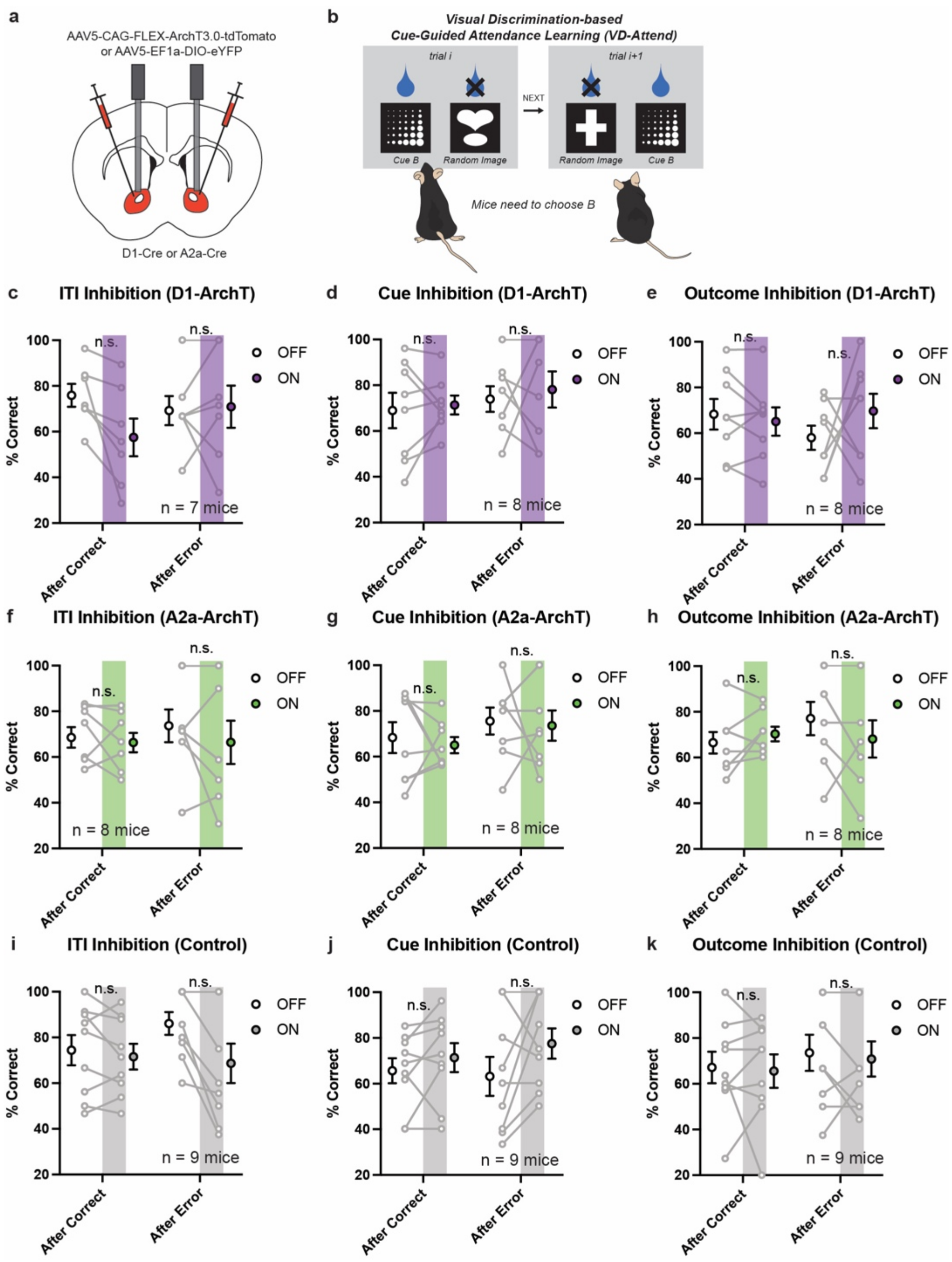

Supplementary Fig. S21. Post-Error Activation of D2-MSNs is Dispensable for Attendance-Based Choice Behavior

(a) Schematic of viral injection and optic fiber implantation. (b) Experimental design. (c-e) Optogenetic suppression of D1-MSNs in the NAc during the ITI (c), Cue (d) or Outcome periods (e) did not affect the behavioral performance of ArchT mice in the next trial (c, Two-way RM-ANOVA with Sidak correction; After Correct, $p = 0.1384$; After Error, $p = 0.9933$; d, Two-way RM-ANOVA with Sidak correction; After Correct, $p = 0.9632$; After Error, $p = 0.8968$; e, Two-way RM-ANOVA with Sidak correction; After Correct, $p = 0.9011$; After Error, $p = 0.3044$). (f-h) Optogenetic suppression of D2-MSNs in the NAc during the ITI (f), Cue (g) or Outcome periods (h) did not affect the behavioral performance of ArchT mice in the next trial (f, Two-way RM-ANOVA with Sidak correction; After Correct, $p = 0.8744$; After Error, $p = 0.3013$; g, Two-way RM-ANOVA with Sidak correction; After Correct, $p = 0.9410$; After Error, $p = 0.9777$; h, Two-way RM-ANOVA with Sidak correction; After Correct, $p = 0.7430$; After Error, $p = 0.2497$). (i-k) Optical stimulation in the NAc during the ITI (i), Cue (j) or Outcome periods (k) did not affect the behavioral performance of ArchT mice in the next trial (i, Two-way RM-ANOVA with Sidak correction; After Correct, $p = 0.9442$; After Error, $p = 0.1382$; j, Two-way RM-ANOVA with Sidak correction; After Correct, $p = 0.5641$; After Error, $p = 0.0675$; k, Two-way RM-ANOVA with Sidak correction, Treatment effects; After Correct, $p = 0.8603$; After Error, $p = 0.8603$). Data are presented as mean ± SEM.
