# Supplementary material for: Error-related signaling in nucleus accumbens D2 receptor-expressing neurons guides inhibition-based choice behavior in mice
Source: Nat Commun. 2023 Apr 21;14:2284. doi: 10.1038/s41467-023-38025-3 (PMC10121661; doi:10.1038/s41467-023-38025-3)
Supplement: Supplementary file 3 — Description of Additional Supplementary Files [file 41467_2023_38025_MOESM3_ESM.pdf]

Description of Additional Supplementary Files

File Name: Supplementary Movie 1
Description: Calcium Imaging In A Correct Trial From A D2-Cre Mouse Performing The VDInhibit task.

File Name: Supplementary Movie 2
Description: Calcium Imaging In An Error Trial From A D2-Cre Mouse Performing the VDInhibit Task.
