# Supplementary material for: Error-related signaling in nucleus accumbens D2 receptor-expressing neurons guides inhibition-based choice behavior in mice
Source: Nat Commun. 2023 Apr 21;14:2284. doi: 10.1038/s41467-023-38025-3 (PMC10121661; doi:10.1038/s41467-023-38025-3)
Supplement: Supplementary file 6 — Reporting Summary [file 41467_2023_38025_MOESM6_ESM.pdf]

nature portfolio

Corresponding author(s): Tadaaki Nishioka, Tom Macpherson, Takatoshi Hikida

Last updated by author(s): Mar 27, 2023

Reporting Summary

Nature Portfolio wishes to improve the reproducibility of the work that we publish. This form provides structure for consistency and transparency in reporting. For further information on Nature Portfolio policies, see our Editorial Policies and the Editorial Policy Checklist.

Statistics

For all statistical analyses, confirm that the following items are present in the figure legend, table legend, main text, or Methods section.

n/a	Confirmed	
☐	☒	The exact sample size (*n*) for each experimental group/condition, given as a discrete number and unit of measurement
☐	☒	A statement on whether measurements were taken from distinct samples or whether the same sample was measured repeatedly
☐	☒	The statistical test(s) used AND whether they are one- or two-sided<br>*Only common tests should be described solely by name; describe more complex techniques in the Methods section.*
☐	☒	A description of all covariates tested
☐	☒	A description of any assumptions or corrections, such as tests of normality and adjustment for multiple comparisons
☐	☒	A full description of the statistical parameters including central tendency (e.g. means) or other basic estimates (e.g. regression coefficient) AND variation (e.g. standard deviation) or associated estimates of uncertainty (e.g. confidence intervals)
☐	☒	For null hypothesis testing, the test statistic (e.g. *F*, *t*, *r*) with confidence intervals, effect sizes, degrees of freedom and *P* value noted<br>*Give P values as exact values whenever suitable.*
☒	☐	For Bayesian analysis, information on the choice of priors and Markov chain Monte Carlo settings
☒	☐	For hierarchical and complex designs, identification of the appropriate level for tests and full reporting of outcomes
☐	☒	Estimates of effect sizes (e.g. Cohen's *d*, Pearson's *r*), indicating how they were calculated

Our web collection on statistics for biologists contains articles on many of the points above.

Software and code

Policy information about availability of computer code

Data collection	ABET II and WhiskerServer software (Lafayette) were used to control operant system and data collection. We acquired fluorescent recordings using commercial software for the Inscopix nVista system.
Data analysis	Data analysis for fluorescent recordings was performed by Inscopix Data Processing software version 1.3.0, ImageJ2 version 2.9.0, and MATLAB_R2022a (Mathworks). Statistical analysis was performed using Prism version 9.5.1 (Graphpad) and MATLAB_R2022a.

For manuscripts utilizing custom algorithms or software that are central to the research but not yet described in published literature, software must be made available to editors and reviewers. We strongly encourage code deposition in a community repository (e.g. GitHub). See the Nature Portfolio guidelines for submitting code & software for further information.

Data

Policy information about availability of data

All manuscripts must include a data availability statement. This statement should provide the following information, where applicable:

- Accession codes, unique identifiers, or web links for publicly available datasets
- A description of any restrictions on data availability
- For clinical datasets or third party data, please ensure that the statement adheres to our policy

Source data are provided with this paper.

March 2021

Human research participants

Policy information about studies involving human research participants and Sex and Gender in Research.

Reporting on sex and gender	N/A
Population characteristics	N/A
Recruitment	N/A
Ethics oversight	N/A

Note that full information on the approval of the study protocol must also be provided in the manuscript.

Field-specific reporting

Please select the one below that is the best fit for your research. If you are not sure, read the appropriate sections before making your selection.

☒ Life sciences ☐ Behavioural & social sciences ☐ Ecological, evolutionary & environmental sciences

For a reference copy of the document with all sections, see nature.com/documents/nr-reporting-summary-flat.pdf

Life sciences study design

All studies must disclose on these points even when the disclosure is negative.

Sample size	No statistical methods were used for predetermining sample sizes, but the sample size was similar to previous studies: see DOI: 10.1016/j.celrep.2020.01.023 and DOI: 10.1126/sciadv.abn3552.
Data exclusions	For optogenetics, two D2-Cre mice were excluded due to insufficient conditioning. For calcium imaging, one D2-Cre mouse was excluded because of incorrect GRIN lens placement.
Replication	All behavioral and imaging data were acquired from a minimum of 2 up to 5 independently performed experimental series. All attempts at replication were successful.
Randomization	C57BL/6J, D1-Cre, D2-Cre, and A2a-Cre mice were assigned randomly to experimental groups.<br>Light stimulation was performed in a random order in 50% of trials. The timing of stimulation (ITI, Cue, or Outcome) was changed for each session, and the order of stimulation timing was randomized among mice.
Blinding	Data collection was not performed blind to the conditions of the experiments. However, we used the same scenarios and custom-written codes for collecting and analyzing data.

Reporting for specific materials, systems and methods

We require information from authors about some types of materials, experimental systems and methods used in many studies. Here, indicate whether each material, system or method listed is relevant to your study. If you are not sure if a list item applies to your research, read the appropriate section before selecting a response.

Materials & experimental systems

n/a	Involved in the study	
☒	☐	Antibodies
☒	☐	Eukaryotic cell lines
☒	☐	Palaeontology and archaeology
☐	☒	Animals and other organisms
☒	☐	Clinical data
☒	☐	Dual use research of concern

Methods

n/a	Involved in the study	
☒	☐	ChIP-seq
☒	☐	Flow cytometry
☒	☐	MRI-based neuroimaging

Animals and other research organisms

Policy information about studies involving animals; ARRIVE guidelines recommended for reporting animal research, and Sex and Gender in Research

Laboratory animals	Wild-type C57BL/6J mice (male, 8-10 weeks old) were used for validation of behavioral experiments. For optogenetic, and calcium imaging experiments, 8-12 weeks old male heterozygous D1-Cre (FK150Gsat), D2-Cre (ER44Gsat) and A2a-Cre 2M strain mice were used.
Wild animals	The study did not involve wild animals.
Reporting on sex	All experiments were performed with male mice.
Field-collected samples	The study did not involve field-collected samples.
Ethics oversight	All experiments conformed to the guidelines of the National Institutes of Health experimental procedures, and were approved by the Animal Experimental Committee of Institute for Protein Research at Osaka University (approval ID 29-02-1 and R04-01-0).

Note that full information on the approval of the study protocol must also be provided in the manuscript.
